# Low immunogenicity of seasonal trivalent influenza vaccine among patients receiving docetaxel for a solid tumour: results of a prospective pilot study

**DOI:** 10.1038/bjc.2011.142

**Published:** 2011-05-03

**Authors:** P Loulergue, J Alexandre, I Iurisci, S Grabar, J Medioni, S Ropert, V Dieras, F Le Chevalier, S Oudard, F Goldwasser, P Lebon, O Launay

**Affiliations:** 1 Université Paris Descartes, Faculté de Médecine, Inserm CIC BT505, Assistance Publique Hôpitaux de Paris (AP-HP), Groupe Hospitalier Cochin Saint Vincent de Paul, CIC de Vaccinologie Cochin-Pasteur, 27 rue du Faubourg Saint Jacques, Paris, 75014, France; 2 Université Paris Descartes, AP-HP, Groupe Hospitalier Cochin Saint Vincent de Paul, Unité d’oncologie médicale, Paris, 75014, France; 3 Institut Curie, Service d’oncologie, Paris, 75005, France; 4 Université Paris Descartes, Faculté de Médecine, AP-HP, Groupe Hospitalier Cochin Saint Vincent de Paul, Service de Biostatistiques, Paris, 75014, France; 5 Université Paris Descartes, AP-HP, Hôpital Européen G Pompidou, Service d’oncologie Médicale, Paris, 75015, France; 6 Université Paris Descartes, AP-HP, Groupe Hospitalier Cochin Saint Vincent de Paul, Laboratoire de Virologie, Paris, 75014, France

**Keywords:** influenza, vaccine, chemotherapy

## Abstract

**Background:**

Patients receiving cytotoxic therapy for solid tumours are at risk of severe influenza. However, few data are available regarding the immunogenical efficacy of influenza vaccine in these patients.

**Methods:**

In this prospective study, 25 patients with breast (*n*=13) or prostate (*n*=12) cancer received a trivalent inactivated influenza vaccine along with docetaxel (Taxotere) administration. The influenza virus type A and B antibody titres were measured using haemagglutinin inhibition (Garten *et al*, 2009) before and 21 days after the vaccination. Seroconversion rate was defined as the percentage of patients with an increase in the serum titres ⩾4 after vaccination.

**Results:**

Median age was 65 years (range: 33–87 years); 52% were females. Seroconversion rates were low: 28% (95% CI: 23.1–33.3) for H1N1, 8% (95% CI: 7.7–8.3) for H3N2 and 16% (95% CI: 7.7–25) for the B strain. The geometric mean titres ratios were 2.16 (H1N1), 1.3 (H3N2) and 1.58 (B). No serious adverse event (AE) related to the vaccine was reported. All the reported AE were from mild-to-moderate intensity.

**Conclusion:**

In the patients receiving docetaxel for solid tumours, influenza vaccine triggers an immune response in only one third. Strategies using more immunogenic influenza vaccines must be evaluated in such patients.

Influenza is a common respiratory infectious disease, which operates in an epidemic mode. It is responsible for secondary bacterial infections of lower respiratory tract causing a sharp increase in morbidity and mortality (Simonsen *et al*, 2000; Thompson *et al*, 2004). Infants, young children, the elderly, pregnant women, but also individuals with chronic disease or underlying immunosuppression are considered at increased risk of death or complications from seasonal influenza (Neuzil *et al*, 2000; Cooksley *et al*, 2005; Fiore *et al*, 2007). In patients with cancer, influenza-associated infections cause mortality estimated at 9%, which is significantly higher than the mortality in the general population. The overall case-fatality rate is considered to be low on average (<0.1%), but is higher in vulnerable populations like elderly people (approaching 1%) and in patients with chronic underlying conditions (Foppa and Hossain, 2008; Nogueira *et al*, 2009). In addition, infectious events may postpone the administration of chemotherapy, lowering dose intensity and thereby being detrimental to the care of cancer patients.

Influenza vaccination is an effective means of preventing influenza and its complications. It allows a reduction of morbidity and mortality secondary to influenza, and is cost-effective in healthy adults (Demicheli *et al*, 2000).

Influenza vaccines are mostly inactivated vaccines, composed of three influenza viruses strains selected annually on the basis of epidemiological data by annual WHO recommendations, two influenza A and one B virus. A vaccine dose contains 15 *μ*g of haemagglutinin for each strain and is given intramuscularly or subcutaneously.

Influenza vaccination is recommended by several health authorities in immunosuppressed patients, including patients receiving chemotherapy (Fiore *et al*, 2007). Despite these recommendations, the rate of influenza vaccination coverage among patients at high risk of complications, including patients with cancer, heart condition, diabetes, chronic respiratory disease or renal failure, is very low: 25% of adults between 18 and 64 years in the United States (18% among 18–49 years to 32% in 50–64 years) (Fiore *et al*, 2007). In France, we recently showed a similar coverage of flu vaccination in patients with cancer (30%) (Loulergue *et al*, 2008). This low rate may be explained by a misunderstanding of the recommendations and concerns of doctors about the efficacy and safety of the influenza vaccine in this population. In industrialised countries, influenza vaccines offer ∼70–90% protection against clinical disease in healthy adults, provided there is a good match between the vaccine antigens and circulating virus(es) (Palache, 1997; Anonymous, 2005). Adults aged over 65 years typically have a diminished immune response to influenza vaccination compared with young healthy adults (Jefferson *et al*, 2010). Response to vaccination is probably reduced as well in patients treated with chemotherapy, but the published data show highly variable results because of the heterogeneity of tumours and treatments (Brydak and Machala, 2000; Brydak *et al*, 2001, 2006). Of note, there are no prospective data on clinical efficacy of influenza vaccination of cancer patients. In 2009, docetaxel (Taxotere, Sanofi-Aventis, Paris, France) was one of the most prescribed chemotherapy, as it is an active treatment in some of the most frequent solid tumours: lung, breast and prostate cancers. Docetaxel is also considered as a significantly immunosuppressive cytotoxic agent (Brain *et al*, 2005).

The aim of this study was to evaluate the immunogenicity and the safety of influenza vaccination in patients receiving chemotherapy with docetaxel.

## Materials and methods

### Study design and population

This prospective pilot study was conducted in four academic centres in France during the 2008–2009 winter season. The main objective of the study was to assess the immunogenicity of one injection of a trivalent inactivated influenza vaccine.

Patients were eligible to participate in this study if they were 18 years or older and had received at least one course of docetaxel chemotherapy for a solid tumour, whatever the stage was. Docetaxel had to be given as a monotherapy, no other cytotoxic drug was allowed. Docetaxel was administered at a dose of 75 mg m^–2^ every 3 weeks. A short treatment with corticosteroids, up to 20 mg of prednisone equivalent a day, given for an anti-emetic purpose was accepted, as well as biotherapies with trastuzumab or bevacizumab. Exclusion criteria included allergy to egg proteins, previous history of allergy to any influenza vaccine component, current acute febrile disease at the time of enrolment or any other immunosuppressive disease (such as HIV infection, renal insufficiency, cirrhosis).

The vaccine was administered on day 1 of docetaxel administration. Blood samples were planned for assessment of haemagglutination-inhibition (Garten *et al*, 2009) antibodies before vaccination, 21 days following vaccination and at month 3. Sera were stored frozen at −20°C until analysed.

Written informed consent was obtained from each patient. The protocol was conducted in accordance with the Declaration of Helsinki and French law for biomedical research, and was approved by the local Ethics Committee (‘Comité de Protection des Personnes Ile-de-France III’, Paris, France).

### Study vaccine

The vaccine was Vaxigrip (Sanofi-Pasteur MSD, Lyon, France), a trivalent inactivated influenza vaccine, in single-dose presentation. The vaccine licensed for the 2008–2009 season contained the A/Solomon Islands/3/2006 (H1N1), A/Wisconsin/67/2005 (H3N2) and B/Malaysia/2506/2004 strains, formulated to contain 15 *μ*g of haemagglutinin antigen of each strain. Vaccines were pre-packaged in 0.5 ml syringes and administered intramuscularly in the deltoid muscle using standard sterile technique.

### Safety surveillance

Patients were observed for a 30-min period after immunisation to monitor for any hypersensitivity reactions or immediate adverse reactions. They were then provided a diary card and were instructed to record the maximum daily measurement or maximum severity of solicited injection site reaction (swelling, tenderness, erythema, induration, redness) and systemic symptoms (fatigue, chills, malaise, myalgia, arthralgia, headache, nausea/vomiting, sweating) for 21 days after vaccination. Patients received the instruction to monitor their body temperature for 7 days twice daily, and report any fever happening between day 7 and day 21. Severity of vaccine-related adverse events (AEs) was graded as mild: mild or transient discomfort, without limitation of normal daily activities; no medical intervention or corrective treatment required; moderate: mild-to-moderate limitation of normal daily activities, minimal medical intervention required; or severe: marked limitation of normal daily activities, medical intervention and corrective treatment required, possible hospitalisation. Erythema and oedema at the site of injection were graded after measurement of local reaction (mild: 0–50 mm; moderate: 50–100 mm; severe: >100 mm). All unsolicited AEs were recorded for 3 months after immunisation and assessed by the investigator for severity and relationship to the study vaccine.

### Laboratory methods

Antibodies against influenza were measured using a standardised HI assay as previously reported (Launay *et al*, 2008; Candon *et al*, 2009). HI was performed in a microtitre test using a 0.5% suspension of O human Rhesus negative erythrocytes and four haemagglutination units of the appropriate antigens. Monovalent haemagglutinating antigens were generously given by Sanofi-Pasteur, Lyon, France, and obtained from viral strains disrupted with detergents. The stains of influenza virus studied were the A/Solomon Islands/3/2006 (H1N1), A/Wisconsin/67/2005 (H3N2) and B/Malaysia/2506/2004. HI assays were performed in duplicate for each sample, using serial twofold dilutions with a starting dilution of the treated serum of 1 : 50 to 1 : 6400. The titration end point is taken as the highest dilution that completely inhibited haemagglutination. The lower limit of quantification was set at 1 : 50. Titres below this level were reported as <1 : 50. All the samples of serum for each patient were analysed for HI antibodies in a same assay.

### Immunogenicity assessment

The primary end point was taken as the immunogenicity observed 21 days post-vaccination. The seroconversion rate was defined as the proportion of patients with a fourfold or more increase HI antibody between baseline and 21; and the seroconversion factor as the fold increase in HI antibody titre post-vaccination (post-vaccination antibody titre divided by the pre-vaccination titre).

To confirm the seroconversion observed by the IHA, immunogenicity was assessed for the H1N1 strain by ELISA. Influenza-specific total IgG was determined using a strain-specific antigen capture ELISA as previously described except that the plates were coated with the detergent disrupted A/Solomon Islands 3/2006 H1N1 strain at a dilution calculated to detect the higher optical density (OD) with the higher dilution of serum. After washing, the coating plates were incubated with 100 *μ*l of patients sera in duplicate, at room temperature for 30 min; after washing, the plates were incubated in the same conditions with 100 *μ*l of a peroxydase-conjugated anti-human IgG (Behring, Marburg, Germany). Plates were washed and 100 *μ*l of colorimetric substrate TMB (Behring) was added for 30 min and the reaction was stopped by the addition of 100 *μ*l of arrest solution according to the manufacturer's instructions. Optical density of the samples was determined at 450 nM. Mean of OD>0.2 between first and second samples was considered as significant.

### Statistical analysis

Descriptive and exploratory analyses were used to evaluate the demographic characteristics stratified by different types of cancer. The frequency and percentage of subjects who had solicited injection site and systemic reactions were calculated. The geometric mean of HI titres (GMT), the seroprotection rate and the seroconversion rate were determined for each of the three influenza vaccine strains. A 95% confidence interval (Cooksley *et al*, 2005) was computed for immunogenicity parameters. Both safety and immunogenicity data were stratified according to cancer group. All comparisons were done using the Pearson's *χ*^2^ test or Fisher's exact test for qualitative variables and the Wilcoxon's test for continuous variables. All tests were two-sided, and *P*-values <0.05 were considered to denote statistical significance. Statistical analyses were performed with SAS software package version 9.2 (SAS Institute, Cary, NC, USA).

## Results

### Subjects

(Table 1) A total of 26 patients were enrolled between 25 September 2008 and 20 January 2009. One patient withdrew his consent and did not received the vaccine; one patient was lost to follow-up after day 1 and did not attend to the two following visits; and two more patients were lost to follow-up after day 21 and did not attend to the third visit (month 3). Finally, 24 subjects were available for the day 21 immunogenicity and safety analyses, and 22 patients for the month 3 analyses. The characteristics of the enrolled population are presented in Table 1.

### Immunogenicity

(Table 2) At baseline, the GMT were ranging from 185.6 (95% CI: 125.7–274.1) for H3N2 to 358 (95% CI: 284.1–451.1) for H1N1. No evidence of higher pre-vaccination titres was evidenced in subject vaccinated against flu in the previous 3 years for any strains titres: 400 *vs* 336 for H1N1 (*P*=0.73), 216 *vs* 138 for H3N2 (*P*=0.21) and 200 *vs* 180 for B (*P*=0.71).

By day 21 after vaccination, the seroconversion rates were 29% (95% CI: 23.1–33.3), 8% (95% CI: 7.7–8.3) and 17% (95% CI: 7.7–25); and the seroconversion factors 2.16 (95% CI: 2.10–2.22), 1.3 (95% CI: 1.26–1.34) and 1.58 (95% CI: 1.45–1.73) for the H1N1, H3N2 and B strains, respectively.

The ELISA tests for H1N1 showed a mean OD at 0.310 at baseline, which increased to 0.481 at day 21. All the seven patients who have responded to the vaccine increasing their titre of inhibiting haemagglutination antibodies have an increase of OD>0.2 between day 1 and day 21 samples (mean OD difference=0.330).

The age of the patients (<65 or >65 years), the level of CD4 T-cell count (<300 or >300 mm^–3^) at the time of vaccination and the previous flu vaccinations were not found as factors influencing the seroconversion in our study. The type of cancer, which is correlated to the use of steroids (as all prostate cancer patients received a 10 mg dose of prednisone daily), seemed to have a role in the response to the B strain: prostate cancer patients had a significantly better response (GMT ratio at day 21) than breast cancer patients (*P*=0.008).

Three months after vaccination, the seroprotection rates were similar to those observed after 3 weeks.

### Safety

Two patients experienced a serious AE none attributable to the vaccine: a hyperalgic low back pain that occurred 14 weeks after vaccination and an obstructive renal failure episode, 12 weeks after vaccination.

No immediate AEs were reported. Nine patients (36%) experienced AEs in the 21 days following the injection. Most of the reported AEs were local: injection site redness (two patients), pain at the injection site (two patients), swelling (one patient), haematoma (one patient). Three patients reported systemic reactions: fever (*n*=1), myalgia (*n*=1) and nausea (*n*=1). The patient who reported fever was a 47-year-old woman followed for a breast cancer. She experienced fever (maximum 38.8°C) during 24 h, beginning 5 days after the immunisation and 1 day after a subcutaneous injection of lenograstim (34 MUI). She did not take any anti-pyretic drug and the fever resolved spontaneously the day after. The link to the vaccine remains unsure. All of those AEs were graded as mild, except one (haematoma at the site of injection) graded as moderate and resolved spontaneously in 1–2 days. No patients declared influenza or influenza-like illness during the study period.

## Discussion

This study showed that one dose of a seasonal inactivated influenza vaccine confers a low immune response in cancer patients receiving docetaxel. Seroconversion rates after vaccination were low, 28% for H1N1, 8% for H3N2 and 16% for the B strain, and the GMT ratios were 2.16 for H1N1, 1.3 for H3N2 and 1.58 for the B strain. There was no difference in the immune responses of patients vaccinated in the 3 years before and those who were not.

Previous studies on the immune response to influenza vaccine in immunosuppressed patients were hampered by small patient numbers, the heterogeneity of the patient population included and the criteria used for the measurement of efficacy (Anderson *et al*, 1999; Brydak and Machala, 2000; Brydak *et al*, 2001, 2006; Ring *et al*, 2002; Vilar-Compte *et al*, 2006). In cancer patients not receiving chemotherapy, the immunogenicity of the influenza vaccine is approaching the level of healthy persons (Anderson *et al*, 1999; Brydak *et al*, 2006). Seroprotection was generally found between 20 and 60% in patients with solid tumours receiving chemotherapy at the time of vaccination (Ramanathan *et al*, 2002). Response rates for influenza vaccination varied between 40 and 80% for breast (Brydak *et al*, 2001; Vilar-Compte *et al*, 2006), colorectal (Puthillath *et al*, 2011) and lung cancer (Anderson *et al*, 1999): when seroconversion rates were the criteria of efficacy, response rate was around 40%, and when seroprotection rate was chosen, the rates increased to 80% (Pollyea *et al*, 2010). There are very few data available for prostate cancer, and we found a low seroconversion rate in our study. The subjects analysed in those studies were not stratified for tumour type or tumour stage, chemotherapy schedule or vaccine type, which makes definite conclusions difficult. However, the studies illustrated that serological responses to different viral antigens might vary considerably between patients and were often inferior to those noted on healthy persons. In a recent meta-analysis on the 76 clinical studies performed between 1982 and 2006 with the trivalent subunit influenza vaccine on healthy subjects, seroconversion rates were found between 60% and 80% for the three strains (Giezeman *et al*, 2009).

The use of steroids was not found as a confounding effect in our study, which is congruent with published data (de Roux *et al*, 2006).

The timing of vaccination and the chemotherapy administration is a critical point (Boehmer *et al*, 2010). In this study, we chose to vaccinate patients on the day they received their chemotherapy for obvious practical reasons, but the best timing for immunisation remains discussed. The only published study dealing with this issue concluded that it was preferable to vaccinate patients between chemotherapy courses: 36 patients, including 20 with solid tumour, were randomised in two groups, one receiving a bivalent whole virus influenza vaccine at the time of administration of chemotherapy and the other at the time at which blood counts were close to their nadir. The seroconversion was better in the group receiving the vaccine after the chemotherapy than the other one (91 *vs* 56% for the solid tumour patients; *P*<0.05) (Ortbals *et al*, 1977). The seroconversion rate for patients vaccinated on the day of the chemotherapy was twice higher than our rate for the H1N1 strain. Those findings should be confirmed on an homogeneous large number of patients and with a subunit inactivated vaccine, as whole virus influenza vaccines are known to be more immunogenic (Hehme *et al*, 2004).

In this study, the seasonal influenza vaccine was well tolerated among cancer patients. All the local or general reactions were grade mild-to-moderate and resolved within 2 days. Although vaccine side-effects might be expected to be tolerated poorly in patients receiving chemotherapy, previous studies having recorded AEs show that the vaccine was well tolerated, particularly concerning fever, which might be confusing in such patients who can develop febrile neutropenia (Ring *et al*, 2002).

The originality of our study was to include patients who had only one type of chemotherapy and perform a prospective study on the immunogenicity and safety of the vaccine in this homogeneous population. The main limit of our study was the small number of patients, due to a high rate of patient refusal (the planned sample size was 40). This acknowledged the fact that influenza vaccine is not accepted among cancer patients, certainly because influenza is not recognised enough as a potential source of complications by both patients and oncologists (Loulergue *et al*, 2008).

Our study was a preliminary research on an important issue. Our results showed that the inactivated trivalent influenza vaccine was safe but induced a low immune response in patients receiving docetaxel, confirming that those patients are very immunosuppressed. Efforts should be made to increase this response as those patients need to be protected against influenza and its consequences. The 2009 influenza pandemic with the variant H1N1 virus has raised the issue of the vaccination of immunosuppressed hosts in an even more accurate way. Using adjuvants, higher dose of antigens or more immunogenic way of vaccination, such as intradermal injection, might constitute appropriate methods to increase the efficacy of influenza vaccines in this population.

## Figures and Tables

**Table 1:** Characteristics of the study population

**Characteristics**	
Number of patients vaccinated	25
Median age, years (min–max)	65 (33–87)
Male gender, *N* (%)	12 (48)
	
*Type of cancer,* N *(%)*	
Breast	13 (52)
Prostate	12 (48)
	
*Performance status,* N *(%)*	
0	9 (36)
1	11 (44)
2	4 (16)
3	1 (4)
Median number of docetaxel courses at the time of enrollment (min–max)	3 (1–11)
	
*Concomitant therapy,* N *(%)*	
Corticosteroids (10 mg predisone equivalent per day)	12 (48)
Hormonotherapy	1 (4)
Radiotherapy	4 (16)
GCSF use	1 (4)
Median CD4 count, cell mm^–3^ (min–max)	280 (120–1790)
	
*Previous influenza vaccination,* N *(%)*	
2007–2008	15 (60)
2006–2007	14 (56)
2005–2006	12 (48)

Abbreviation: GCSF=granulocyte colony stimulating factor.

**Table 2:** Immune response: inhibiting haemagglutination antibodies against A/Solomon Islands/3/2006 (H1N1), A/Wisconsin/67/2005 (H3N2) and B/Malaysia/2506/2004 strains

	**A/H1/Solomon Islands/3/06**	**A/H3/Wisconsin/67/05**	**B/Malaysia/2506/04**
*Pre-vaccination at day 0*			
Number of tested patients	25	25	25
Geometric mean titre (95% CI)	358 (284.1–451.1)	185.6 (125.7–274.1)	195.4 (151.9–251.5)
			
*Post-dose 1 at day 21*			
Number of tested patients	24	24	24
Geometric mean titre (95% CI)	794.4 (609.4–1036)	257.2 (169.3–390.5)	315.2 (221.40–448.9)
Seroconversion rate, *n* (%) (95% CI)	7 (29) (23.1–33.3)	2 (8) (7.7–8.3)	4 (17) (7.7–25)
Seroconversion factor (95% CI)	2.16 (2.10–2.22)	1.3 (1.26–1.34)	1.58 (1.45–1.73)

Abbreviation: CI=confidence interval.

Seroconversion rate as percentage of patients showing a significant increase in antibody titre defined as a pre-vaccination titre ⩾1 : 50 and at least a fourfold increase in post-vaccination titre. Seroconversion factor or geometric mean fold rise as the geometric mean of the within-subject ratios of the post-vaccination reciprocal HI titre to the day 1 reciprocal HI titre.
